# Clustering of Hypoglycemia Events in Patients With Hyperinsulinism: Extension of the Digital Phenotype Through Retrospective Data Analysis

**DOI:** 10.2196/26957

**Published:** 2021-10-29

**Authors:** Chris Worth, Simon Harper, Maria Salomon-Estebanez, Elaine O'Shea, Paul W Nutter, Mark J Dunne, Indraneel Banerjee

**Affiliations:** 1 Department of Paediatric Endocrinology Royal Manchester Children's Hospital Manchester United Kingdom; 2 Department of Computer Science University of Manchester Manchester United Kingdom; 3 Faculty of Biology, Medicine and Health University of Manchester Manchester United Kingdom

**Keywords:** hyperinsulinism, continuous glucose monitoring, digital phenotype, hypoglycemia, nocturnal hypoglycemia

## Abstract

**Background:**

Hyperinsulinism (HI) due to excess and dysregulated insulin secretion is the most common cause of severe and recurrent hypoglycemia in childhood. High cerebral glucose use in the early hours results in a high risk of hypoglycemia in people with diabetes and carries a significant risk of brain injury. Prevention of hypoglycemia is the cornerstone of the management of HI, but the risk of hypoglycemia at night or the timing of hypoglycemia in children with HI has not been studied; thus, the digital phenotype remains incomplete and management suboptimal.

**Objective:**

This study aims to quantify the timing of hypoglycemia in patients with HI to describe glycemic variability and to extend the digital phenotype. This will facilitate future work using computational modeling to enable behavior change and reduce exposure of patients with HI to injurious hypoglycemic events.

**Methods:**

Patients underwent continuous glucose monitoring (CGM) with a Dexcom G4 or G6 CGM device as part of their clinical assessment for either HI (N=23) or idiopathic ketotic hypoglycemia (IKH; N=24). The CGM data were analyzed for temporal trends. Hypoglycemia was defined as glucose levels <3.5 mmol/L.

**Results:**

A total of 449 hypoglycemic events totaling 15,610 minutes were captured over 237 days from 47 patients (29 males; mean age 70 months, SD 53). The mean length of hypoglycemic events was 35 minutes. There was a clear tendency for hypoglycemia in the early hours (3-7 AM), particularly for patients with HI older than 10 months who experienced hypoglycemia 7.6% (1480/19,370 minutes) of time in this period compared with 2.6% (2405/92,840 minutes) of time outside this period (*P*<.001). This tendency was less pronounced in patients with HI who were younger than 10 months, patients with a negative genetic test result, and patients with IKH. Despite real-time CGM, there were 42 hypoglycemic events from 13 separate patients with HI lasting >30 minutes.

**Conclusions:**

This is the first study to have taken the first step in extending the digital phenotype of HI by describing the glycemic trends and identifying the timing of hypoglycemia measured by CGM. We have identified the early hours as a time of high hypoglycemia risk for patients with HI and demonstrated that simple provision of CGM data to patients is not sufficient to eliminate hypoglycemia. Future work in HI should concentrate on the early hours as a period of high risk for hypoglycemia and must target personalized hypoglycemia predictions. Focus must move to the human-computer interaction as an aspect of the digital phenotype that is susceptible to change rather than simple mathematical modeling to produce small improvements in hypoglycemia prediction accuracy.

## Introduction

### Background

Hyperinsulinism (HI) is a diverse collection of disorders united by the pathology of inappropriate insulin secretion causing hyperinsulinemic hypoglycemia with simultaneous suppression of alternative fuel sources. It has an estimated incidence of 1:28,000 in the United Kingdom [1] and is the most common cause of severe and recurrent hypoglycemia in childhood. This recurrent hypoglycemia, with the corresponding suppression of ketones as an alternative fuel source, results in brain damage in an unacceptably high proportion of cases, up to 48% [2]. The risk of brain damage is independent of the chronicity of the disease [3], as damage often occurs early in life when the neonatal brain is highly susceptible to such insults [4,5].

Significant progress in the understanding of the underlying pathophysiology of HI has been made since its first detailed description in 1953 [6]. Increased knowledge of changes at the organic [7], cellular [8], and genetic [9,10] levels has led to improvements in the care of these patients, and recent studies even suggest a lowering of the subsequent rate of brain injury [11]. As first suggested by Richard Dawkins in 1976, there is an “extended phenotype” of all conditions, not just limited to observable physical traits or cellular changes [12]. The most recent extension of this is that of a digital phenotype, encompassing aspects of patients’ behaviors related to and measured by technology [13]. The digital phenotype includes everything from interactions with others on social media to digitally collected location data and continuously measured physiological parameters, such as glucose levels and heart rate. These measures sit alongside the traditional characterization of diseases to form a more comprehensive picture and facilitate a more nuanced approach to management. Current management of HI is complex, balancing the risks and benefits of a limited repertoire of medications, all of which have a small therapeutic window and significant side effect profiles [14]. Because of the dysregulated secretion of insulin in these patients, hypoglycemic events are often very difficult to predict. The standard of care for home monitoring of hypoglycemia is intermittent fingerpick testing for blood glucose. However, patients rarely achieve more than three to four such measurements per day and this infrequent testing strategy risks missing hypoglycemia between tests, particularly overnight [15]. This practice also offers little in the way of disease characterization and does very little to extend the phenotype or the scientific understanding of HI.

In recent years, continuous glucose monitoring (CGM) has emerged as an alternative, offering insight into glucose trends. CGM measures subcutaneous glucose at frequent intervals over extended periods (7-10 days) to provide glycemic phenotypes in patients with hypoglycemia and diabetes and contributes to the digital phenotype [16]. The application of CGM may not yet be readily applicable in patients with HI as a reliable means of hypoglycemia detection or prediction [17]. However, as CGM is a passive form of monitoring, it can record data at a high granularity with very minimal response burden on users [18]; therefore, it has the potential to collect detailed glycemic data while being acceptable to patients on a long-term basis. There have been a limited number of studies describing the utility of CGM in patients with HI [19-22], and none have described the timing of hypoglycemia events or glycemic trends.

There is good empirical evidence [23] to suggest that hypoglycemic events may not be evenly distributed throughout the day and that the risk of hypoglycemia may be disproportionately higher during periods of reduced food intake. In all but the youngest children, each day is divided into two distinct phases, one of activity and eating and the other of fasting and rest [23]. It is well established that hormones such as cortisol vary throughout the day, with peak levels varying in relation to the time of sunrise [24]. Glucose homeostasis also varies with the time of day [25], but this variability is not likely to be directly related to cortisol [26,27]. Rather, there is likely a direct circadian control involving the suprachiasmatic nucleus in the hypothalamus [28,29] and peripheral clock-gene–regulated components in the pancreas [23].

Before waking, early morning is a high-risk period for hypoglycemia, as glucose use is at its highest [23]. Normally, this is counteracted by a high rate of gluconeogenesis [23]; however, in patients with HI, this is suppressed owing to inappropriate secretion of insulin, resulting in an imbalance of glucose homeostasis weighted toward hypoglycemia [30]. The high risk in early morning is exacerbated by the time-independent, high-glucose requirement of the brain during late sleep [31].

Despite over 30 years of investigation into nocturnal hypoglycemia in children with diabetes [32], the estimated incidence remains as high as 68% [33]. More than half of all severe hypoglycemic events occur overnight [34], and up to 18% of deaths in younger patients with diabetes are attributed to nocturnal hypoglycemia [35].

The potential for CGM data to contribute to the digital phenotype of HI has not yet been investigated. Despite the physiological and empirical evidence from healthy participants and participants with diabetes regarding the risks of nocturnal hypoglycemia, no study has investigated the timing of hypoglycemic events in children with HI. It is important to identify periods of greater risk of hypoglycemia to design targeted detection, prediction, and prevention strategies, as traditional medical management techniques do not allow for this. Hall et al [36], Colas et al [37], and Lunt et al [38] used CGM as a short-term phenotyping tool to better understand patient profiles and categorize risks. Larkin et al [39] detailed their intention to use CGM as part of a long-term phenotyping tool on a large scale to provide personalized insights into disease.

Digital phenotyping allows for two important changes in disease management. First, the detailed analysis of CGM data provides an extension of the digital phenotype for both the disease and the individual and allows for the targeting of interventions to times when they will achieve the optimum effect. Second, patients and parents are not passive bystanders in the management of HI, and an analysis of how they interact with and respond to the technology further extends the digital phenotype [40], as well as enabling and enhancing behavior change [41]. Knowledge of an extended digital phenotype will not, in and of itself, improve outcomes but does improve understanding of how future interventions can be adapted to achieve the most significant and lasting behavior change [42].

### Objective

In this study, we take the first step in extending the digital phenotype of HI by describing glycemic trends and identifying the timing of hypoglycemia measured by CGM. The following findings provide a basis for future work concentrating on using the newly extended digital phenotype with human-computer interactions and ultimately altering care behaviors of parents to reduce the incidence of damaging hypoglycemia. Our original code is provided as an appendix (Multimedia Appendix 1) and is freely available on GitHub [43].

## Methods

### Recruitment

Patients were recruited between July 2017 and October 2020. Three distinct groups of patients were enrolled in the study: (1) those with a diagnosis of HI who were in hospital for acute management (n=6), (2) those in an outpatient setting with a diagnosis of HI and for whom glycemic control was suboptimal (n=17), and (3) those with a diagnosis of idiopathic ketotic hypoglycemia (IKH) for whom glycemic control was unstable (n=24). The IKH group was selected to investigate CGM profiles in an alternative clinical model of hypoglycemia that does not involve excess insulin secretion. All patients underwent CGM with the primary intention of better understanding their glucose control for clinical purposes. All patients were approached by the research team to seek consent to use anonymized CGM data for research purposes, as per a local research ethics protocol (REC/H1010/88).

Within the HI group, further subcategorization was done as either diffuse or focal disease [44]. Focal HI is characterized by the formation of a focal lesion within the pancreas comprising hyperfunctioning islets and is potentially curative by focal lesionectomy. Diffuse HI implies some specific histopathological features but practically implies nonfocal HI in which medical therapy should be prioritized over surgery when possible.

Patient recruitment to the study was based on a pragmatic design in the absence of previous studies to determine sample size in a rare disease. Previous studies investigating CGM in patients with HI recruited 11 to 15 patients [19,21]. We recruited all patients undergoing CGM for clinical reasons over a 3-year period.

### Data collection

Patients undergoing inpatient monitoring had their CGM device (Dexcom G4 or G6 depending on the date) attached between 1 and 5 days before pancreatic surgery (lesionectomy or subtotal pancreatectomy) and removed after plasma glucose levels stabilized, reducing the need for frequent monitoring. All other patients were brought to the Royal Manchester Children’s hospital to have their CGM device attached by a specialist nurse, after which they returned home for the remainder of the monitoring period. The patients returned CGM devices to the department at the end of the monitoring period. CGM devices were always inserted in the daytime to ensure that full calibration had occurred before the evening and data collected overnight were reliable. Data were collected for between 4 and 10 days from each patient.

For patients who underwent a controlled fast in hospital (n=0 for HI, n=2 for IKH) during CGM, data during the fasting period were deleted from the analysis and the only data used were those acquired from home monitoring after discharge from hospital. Dexcom G4 devices were used from the beginning of the study period until March 2019, from which point all patients were monitored using a Dexcom G6 device. All devices were unblinded so that parents and staff could see glucose values in real time and alarms would sound if glucose levels dropped (or was predicted to drop) below 3.5 mmol/L for those with HI and 3.3 mmol/L for those with IKH (as per lowest allowable device settings). The fall rate and urgent low soon alarms were set to *on* when the device was given to the patient. Patient modifications of the alarms were not routinely investigated.

### Data Analysis

Hypoglycemia was defined as any glucose value <3.5 mmol/L (63 mg/dL) as a safe cutoff used by most specialized centers for the everyday management of HI [14]. Dexcom CGM devices report a glucose value every 5 minutes, and therefore, hypoglycemia events were measured at 5-minute intervals with a minimum duration of 5 minutes. The term *early hours* is used throughout the manuscript to refer to the period 3 AM to 7 AM.

Data were downloaded from CGM devices to Dexcom CLARITY database and raw data were downloaded for analysis using Python 3.8. We analyzed the total number of hypoglycemia events by start time irrespective of length and separately the total amount of time spent in hypoglycemia within each hour period.

As data were not normally distributed, nonparametric tests (Mann–Whitney *U*) were used to assess for differences between continuous variables. Chi-square tests were used to assess differences in proportions or percentages between the groups. The results provided are raw test statistics and associated *P* values.

Glucose testing using alternative methods (plasma glucose measured by point-of-care testing or handheld home glucose monitoring) was not routinely performed alongside CGM because assessment of CGM accuracy was not the intention or focus of this study. Correlations between CGM-derived subcutaneous glucose and plasma glucose have been previously reported in patients with HI [19,21].

## Results

### Overview

Baseline data showed a male predominance (29 males, 18 females) and mean age in months at the time of CGM was higher in those with IKH than in those with HI (82, SD 43 vs 57, SD 61; *P*=.03), as expected, given that HI is prevalent at a younger age than IKH. At the time of CGM testing, the mean time in months from diagnosis did not differ significantly between those with IKH and HI (33, SD 34 vs 49, SD 53; *P*=.43) (Table 1). A total of 28 patients underwent monitoring with a Dexcom G4 device and 19 with a Dexcom G6.

**Table 1:** Demographics of hyperinsulinism and idiopathic ketotic hypoglycemia groups.

Demographics	Hyperinsulinism (N=23)	IKH^a^ (N=24)	*P* value^b^
Male, n (%)	17 (74)	12 (50)	.09
Female, n (%)	6 (26)	12 (50)	N/A^c^
Age (months), mean (SD)	57 (60)	82 (43)	.03
Time since diagnosis (months), mean (SD)	49 (52)	33 (44)	.43

^a^IKH: idiopathic ketotic hypoglycemia.

^b^*P* value for difference between groups calculated via chi square test for sex and Mann–Whitney *U* test for continuous values.

^c^N/A: not applicable.

Demographic data demonstrate that the only difference between groups at baseline was that patients with IKH had a higher mean age at time of CGM.

**Figure 1:**
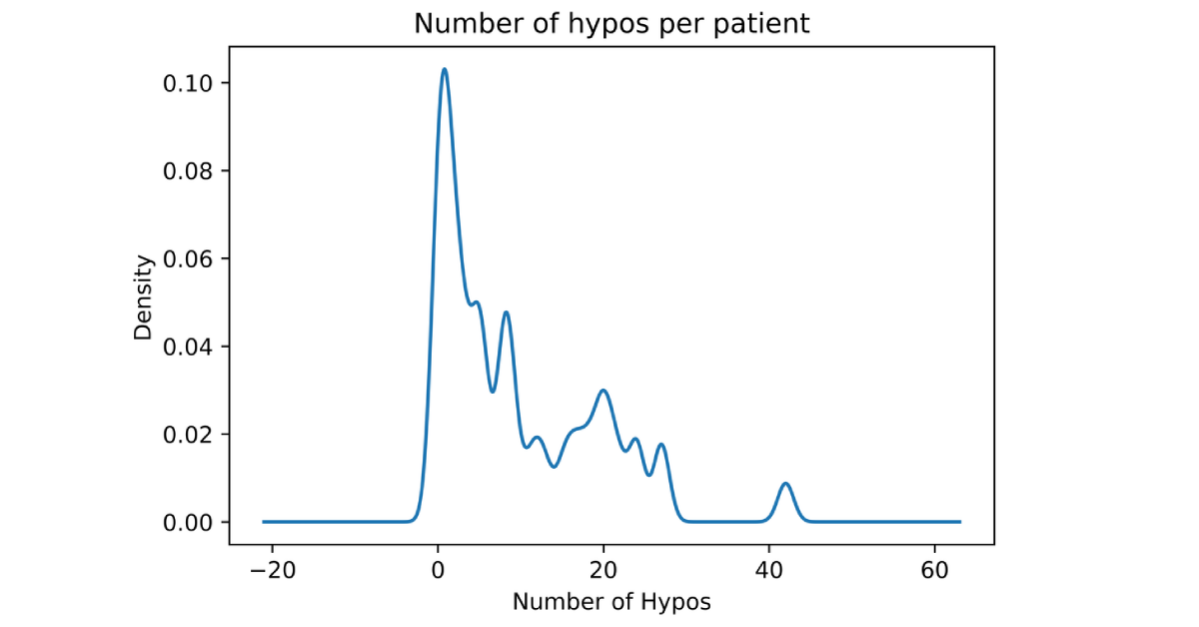
Frequency plot of number of hypoglycemia events (hypos) per patient. This plot demonstrates the positive skew to the distribution of hypoglycemic events. Mean number of hypoglycemia events was 9.5 per patient, and median was 6.0 with 6 patients having no episodes of hypoglycemia and 1 patient having more than 30 separate hypoglycemic events. The majority of patients had 5 to 20 hypoglycemic events.

A total of 449 hypoglycemic events (189 in HI and 260 in IKH) were captured over 237 days. The time spent in hypoglycemia was 15,610 out of a total of 342,355 minutes (4.6%). The mean duration of hypoglycemic events was 35 (SD 57) minutes and was longer in those with IKH than in those with HI (40 vs 28). Mean lowest glucose per hypoglycemia event was 3.1 (SD 0.37) mmol/L. The mean number of hypoglycemic events per patient was 9.5 (SD 9.6) with a positive skew to the distribution (Figure 1), illustrating the small number of patients with a very large number of hypoglycemic events. In patients with HI, there were 42 hypoglycemia events lasting more than 30 minutes from 13 separate patients. The mean duration of such prolonged hypoglycemia events was 79 (SD 72) minutes, with a mean lowest glucose of 2.8 mmol/L. The characteristics of patients with HI are shown in Table 2.

**Table 2:** Characteristics of patients with hyperinsulinism included in the study.^a^

	Sex	Mutation	Type	Surgery	Age (months)	Location	Rx^b^
1	Male	Not done	Diffuse	None	88	Outpatient	None
2	Male	Not done	Diffuse	None	8	Outpatient	None
3	Female	Negative	Diffuse	None	92	Outpatient	None
4	Male	*SLC16A1*	Diffuse	None	34	Outpatient	CHO^c^
5	Male	*SLC16A1*	Diffuse	None	37	Outpatient	CHO^c^
6	Female	Negative	Diffuse	None	119	Outpatient	None
7	Male	*ABCC8*	Diffuse	None	58	Outpatient	Diazoxide
8	Male	*GCK*	Diffuse	None	190	Outpatient	Diazoxide
9	Male	*ABCC8*	Diffuse	Subtotal^d^	141	Outpatient	None
10	Male	*ABCC8*	Diffuse	Subtotal^d^	132	Outpatient	None
11	Male	*ABCC8*	Focal	Lesionectomy	2	Inpatient	Octreotide
12	Male	*ABCC8*	Diffuse	None	36	Outpatient	None
13	Female	*ABCC8*	Diffuse	Subtotal^d^	51	Outpatient	Octreotide
14	Male	*ABCC8*	Focal	Lesionectomy	36	Outpatient	Octreotide
15	Female	Negative	Diffuse	None	17	Outpatient	Diazoxide
16	Female	*ABCC8*	Focal	Lesionectomy	3	Inpatient	Octreotide
17	Male	Negative	Diffuse	None	63	Outpatient	Diazoxide
18	Male	Negative	Diffuse	None	10	Outpatient	Diazoxide
19	Male	*ABCC8*	Diffuse	Subtotal^d^	193	Outpatient	None
20	Male	*ABCC8*	Focal	Lesionectomy	1	Inpatient	None
21	Male	*ABCC8*	Focal	Lesionectomy	3	Inpatient	Octreotide
22	Female	*ABCC8*	Focal	Lesionectomy	1	Inpatient	Octreotide
23	Male	*ABCC8*	Focal	Lesionectomy	3	Inpatient	Octreotide

^a^Important characteristics relating to hyperinsulinism and continuous glucose monitoring are listed for all hyperinsulinism patients individually.

^b^Rx: medical treatment at the time of continuous glucose monitoring.

^c^CHO: carbohydrate supplementation in feeds.

^d^Subtotal: subtotal pancreatectomy.

### Timings of Hypoglycemia in Patients With HI

**Figure 2:**
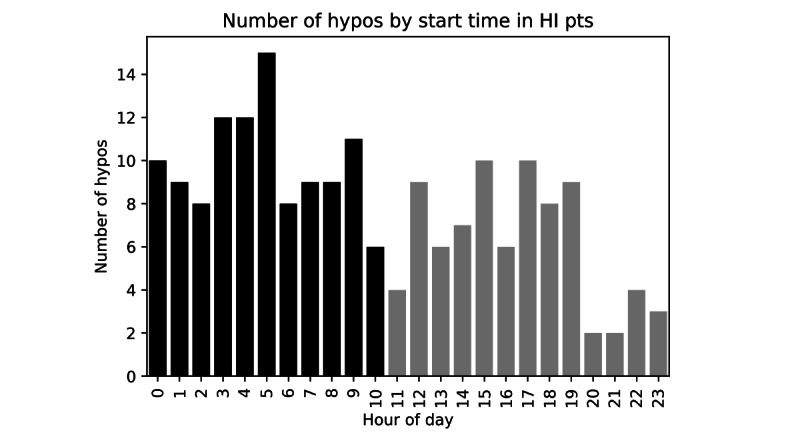
Number of hypoglycemic events (hypos) plotted by start time in patients with HI. The X-axis represents hours of each 24 hour period. Bars represent the number of hypoglycemic events starting at any particular point in the day but do not indicate the duration of each episode. What is demonstrated is the increased number of hypoglycemic episodes starting in the later hours of the night and early morning (black) compared with the rest of the day (grey). HI: hyperinsulinism.

**Figure 3:**
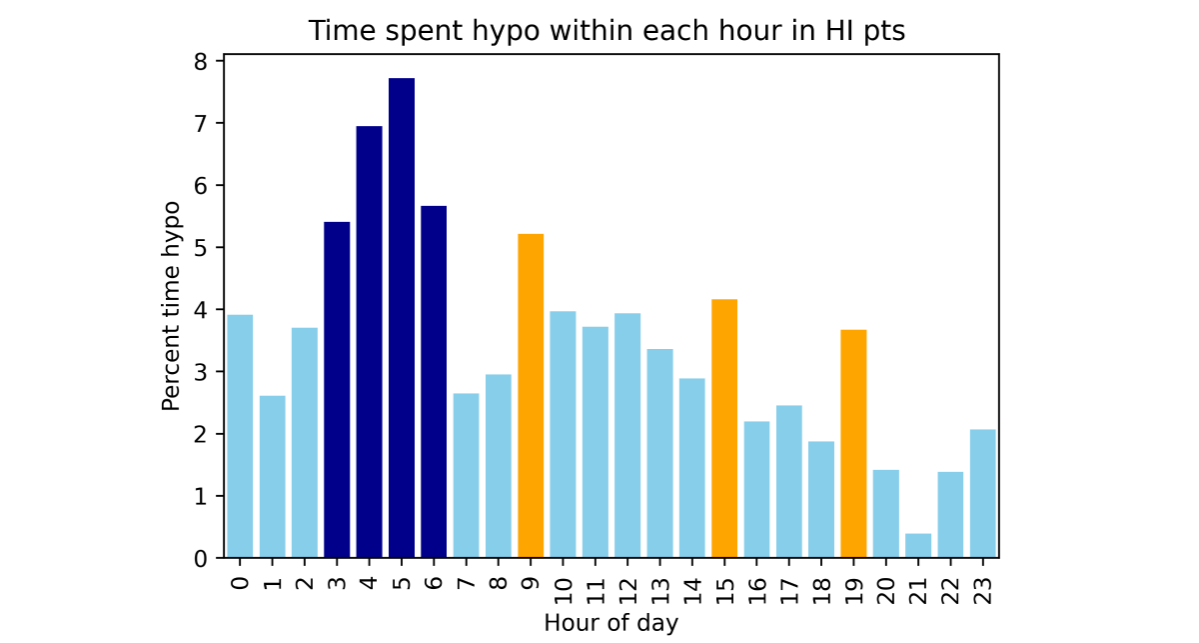
Percentage time spent in hypoglycemia by hour of the day in patients with HI.There is a clear period of high risk for hypoglycemia between 3 AM and 7 AM (dark blue) which represents the early hours. There are also three distinct spikes of increased hypoglycemia prevalence at 9 AM, 3 PM and 7 PM (orange), which may represent postprandial hypoglycemia. HI: hyperinsulinism.

Figure 2 presents the number of hypoglycemic events by start time in patients with HI (n=23). This does not account for the duration of hypoglycemia episodes and is only representative of the hour in which the event started. There was a higher risk of a hypoglycemia event beginning in the later part of the night/early morning compared with the rest of the day. Figure 3 illustrates the percentage of time spent in hypoglycemia by patients with HI within each hour of the day. There was an increase in the prevalence of hypoglycemia in the early hours (3 AM-7AM), 6.4% (1665/25,875 minutes) of this time hypoglycemic compared with only 2.9% (3585/123,490 minutes) of time outside this period (*χ*^2^_1_=98.4, *P*<.001). This represents a doubling of risk during this period. Although the frequency of hypoglycemia was greater in the early hours, the mean duration (minutes) of individual hypoglycemic episodes in this period was the same as the rest of the day (28.2 minutes vs 28.1 minutes, *P*=.99).

The other periods of increased risk were the separate hours of 9 AM, 3 PM, and 7 PM, within which there was a higher proportion of minutes spent hypoglycemic than the rest of the daytime/evening (7 AM to midnight), that is 5.2% (320/6140 minutes), 4.2% (250/6015 minutes), and 3.7% (230/6265 minutes), respectively, versus 2.8% (2915/103,910 minutes; *P*<.001).

### Subgroup Analysis of Patients With HI

**Figure 4:**
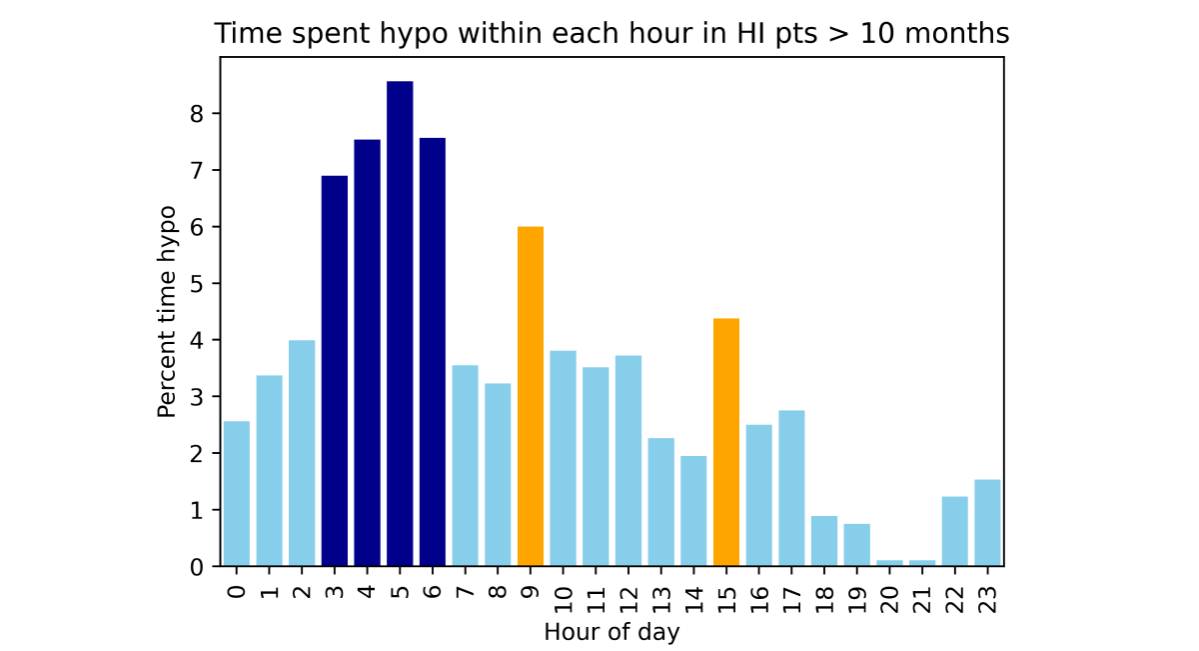
Percentage time spent hypoglycemic by hour of the day in patients with HI > 10 months of age. Analysis of timings of hypoglycemia in this subgroup show a greater tendency to early hours hypoglycemia between the hours of 3 AM and 7 PM (dark blue) with a persistence of spikes in hypoglycemia risk at 9 AM and 3 PM (orange). No spike is observed at 7 PM in contrast to the analysis for all ages. HI: hyperinsulinism.

**Figure 5:**
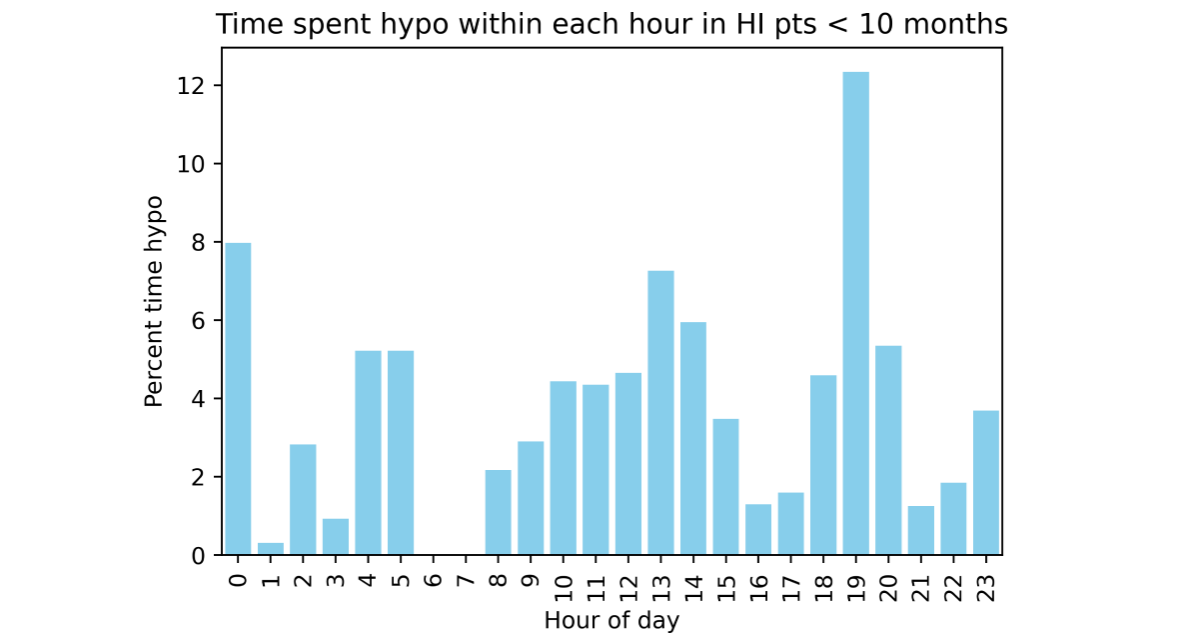
Percentage time spent hypoglycemic by hour of the day in patients with HI < 10 months of age. Analysis of timings of hypoglycemia in this subgroup are limited by numbers but clearly show a very different pattern of hypoglycemia compared with the group > 10 months of age. There is no obvious pattern of hypoglycemia and no obvious periods of higher risk. HI: hyperinsulinism.

Analysis of time spent in hypoglycemia by patients with HI above (n=16) and below (n=7) the age of 10 months (ie, the age at which a weaning diet with solid food is well established) demonstrated that the risk of early hours of hypoglycemia was even more pronounced in the group above the age of 10 months (Figure 4). Within the early hours, 7.6% (1480/19,370 minutes) of the time was hypoglycemic compared with 2.6% (2405/92,840 minutes) of time outside of this period (*χ*^2^_1_=146.4, *P*<.001), indicating an almost trebling risk of hypoglycemia. In the group below the age of 10 months, no obvious patterns of hypoglycemia were visible with risk of hypoglycemia distributed randomly throughout the day, except for an unexplained peak of risk at 7 PM (Figure 5).

Further comparisons were performed between the following HI subgroups: mutation-positive versus mutation-negative and medication versus off-medication. The results are summarized in Table 3 and reported in more detail (along with Figures S1-S4) in Multimedia Appendix 2: further HI subgroup comparisons.

**Table 3:** Comparisons of timings of hypoglycemia between hyperinsulinism (HI) subgroups.^a^

			Time hypoglycemic in early hours (minutes)				Time hypoglycemic outside early hours (minutes)					Minutes hypo contained in early hours (expected 16.7%; minutes)					Period of risk (compared with all HI)
			Total, N		Value, n (%)		Total, N		Value, n (%)		Total, N			Value, n (%)			
All HI			25,875		1665 (6.43)		123,490		3585 (2.9)		5250			1665 (31.71)		Early hours	
**Age (months), mean (SD)**																	
	>10	19,370		1480 (7.64)		92,840		2405 (2.59)		3885			1480 (38.09)		Early hours ++^b^		
	<10	6505		185 (2.84)		37,150		1365 (3.85)		1365			185 (13.55)		Evenly distributed		
**HI causing mutation**																	
	Positive	17,005		1135 (6.67)		81,810		2290 (2.79)		3425			1135 (33.14)		Early hours +^c^		
	Negative	8870		530 (5.98)		41,680		1295 (3.11)		1825			530 (29.04)		Early hours −^d^		
**Medication**																	
	On	14,415		790 (5.48)		69,485		2395 (3.45)		3185			790 (24.8)		Early Hours −−^e^		
	Off	11,460		875 (7.64)		54,005		1190 (2.2)		2065			875 (42.37)		Early Hours ++		

^a^Detailed is the difference in percentage time hypoglycemic in the early hours (3 AM-7 AM). Also reported is the percentage of all hypoglycemia minutes spent in the early hours as a comparison to the expected 16.7% that would be seen if hypoglycemia was distributed evenly. The table demonstrates an exaggerated tendency to hypoglycemia at early hours in those above the age of 10 months, off medication, and with a known hyperinsulinism causing mutation.

^b^++ is used to denote a very strong tendency to early hours hypoglycemia.

^c^+ is used to denote a strong tendency to early hours hypoglycemia.

^d^− is used to denote a relatively weak tendency to early hours hypoglycemia.

^e^−− is used to denote a weak tendency to early hours hypoglycemia.

### Timings of Hypoglycemia in Those With IKH

**Figure 6:**
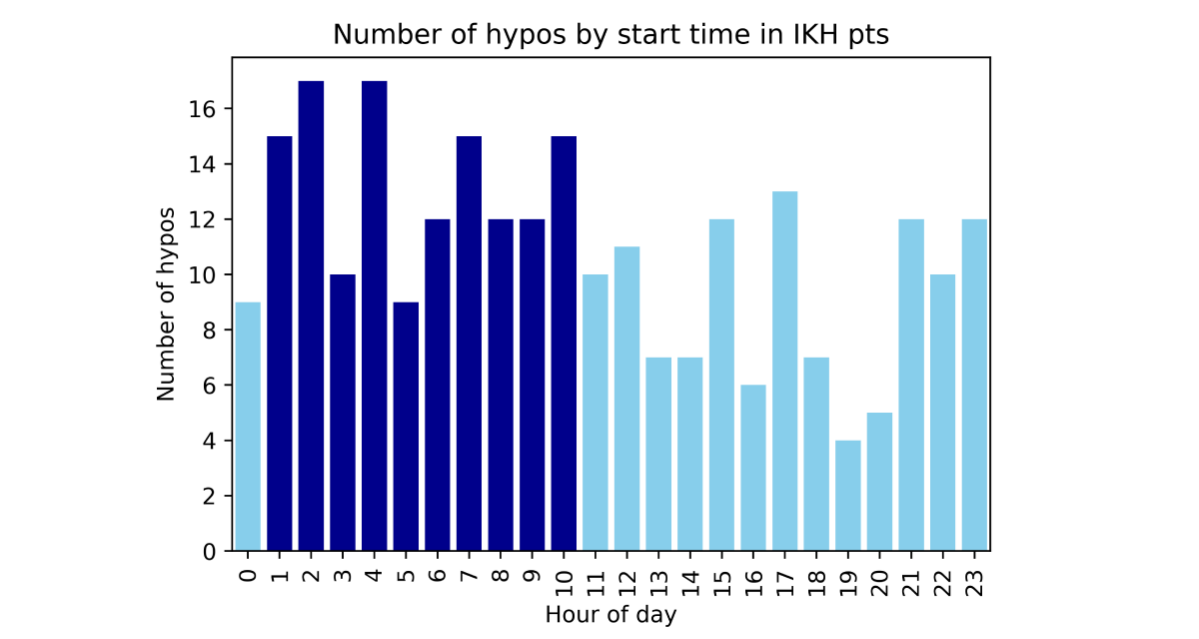
Number of hypoglycemic events (hypos) plotted by hour of the day in patients with IKH. This demonstrates the risk of a hypoglycemic event starting at any particular point in the day but does not account for the length of this episode. An increased number of hypoglycemic episodes starting in the later hours of the night and early morning (dark blue) is observed, compared with the rest of the day (light blue). This, however, is less pronounced than in those patients with HI. HI: hyperinsulinism; IKH: idiopathic ketotic hypoglycemia.

**Figure 7:**
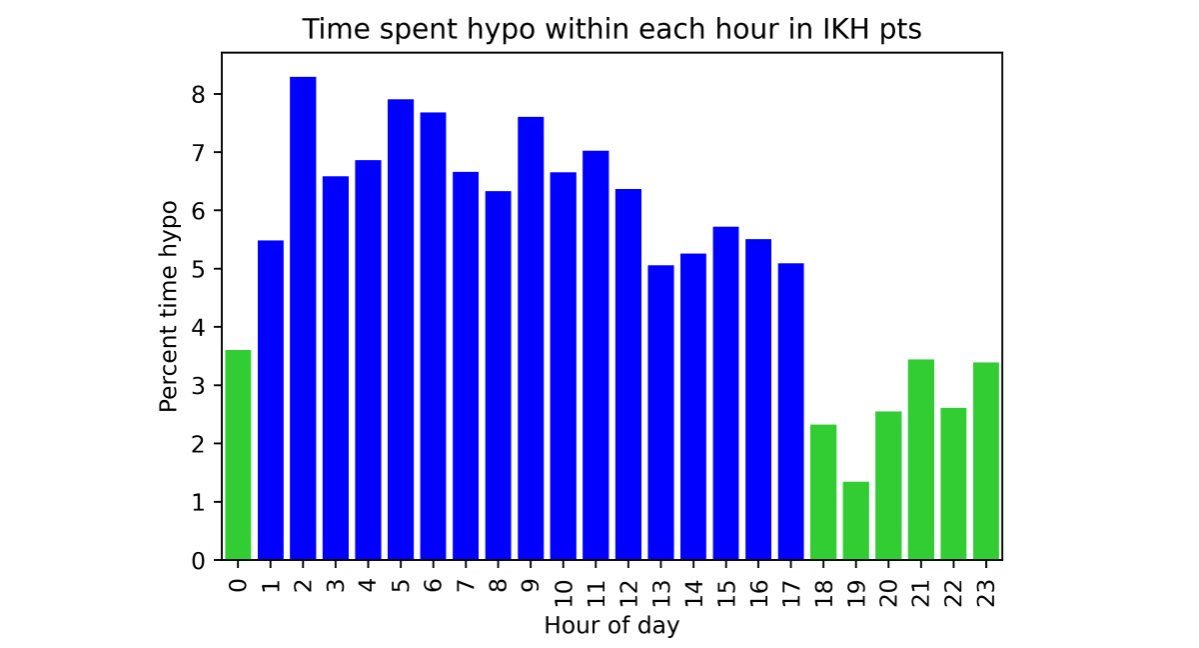
Percentage time hypoglycemic by hour of the day in patients with IKH. There is no period of particularly high risk as seen in patients with HI. In contrast, a short period of lower than average risk in the evening and early night (green) can be observed. HI: hyperinsulinism; IKH: idiopathic ketotic hypoglycemia.

The timing of hypoglycemia in patients with IKH showed a more evenly distributed pattern than in those with HI. The number of hypoglycemic episodes starting overnight was slightly higher than that during the day (Figure 6), but not as markedly as in the patients with HI. Total minutes spent hypoglycemic were also more evenly distributed throughout the day in patients with IKH than in those with HI (Figure 7). There was no clear period of higher than average risk of hypoglycemia; rather, a period of relatively low risk was observed in the evening and early night (6 PM-1 AM). Within this period, the risk of hypoglycemia was 2.8% (1575/57,195 minutes) compared with 6.5% (8785/135,805 minutes) outside of this period (*Χ*^2^_1_=132.2, *P*<.001).

Subgroup analysis of patients with IKH was not performed, as no patients within this group were below the age of 10 months and this group was primarily analyzed as a comparison group for those with HI.

### Altering Thresholds for Hypoglycemia

As the definition of hypoglycemia differs between countries, regions, and even hospital departments, we performed further analysis by altering the threshold for hypoglycemia. The cutoffs of 3.9 mmol/L, 3.5 mmol/L, and 3.0 mmol/L were chosen as commonly used definitions for hypoglycemia in children [44,45]. Further analysis of values below this were deemed unlikely to be meaningful as they are rare events, and the Dexcom CGM devices only measure glucose down to 2.2 mmol/L, below which values are reported as *Low*.

Data for HI patients above the age of 10 months were compared with those of patients with IKH, as these 2 groups were more comparable in terms of age. As hypoglycemia threshold was reduced from 3.9 mmol/L to 3.5 mmol/L and then 3.0 mmol/L, the tendency to hypoglycemia during early hours was emphasized in patients with HI. In patients with HI, the early hours contained 41% of all minutes (63/152 minutes) spent with a glucose level <3.0 mmol/L despite this period representing only 16.7% of the 24-hour period. A similar trend began to emerge in patients with IKH when the hypoglycemia threshold was reduced, and the proportion of all minutes spent hypoglycemic that lay within the early hours increased from 21% (842/3998 minutes) to 27% (157/577 minutes). Figures S5 and S6 provide a visualisation of this change and are provided in Multimedia Appendix 3.

## Discussion

### Principal Findings

We have provided a novel analysis of the timing of hypoglycemic events in patients with HI using CGM data. Our data provide new and clinically useful information to extend the digital phenotype of HI. This aspect of the newly described digital phenotype demonstrates a tendency for hypoglycemia during the early hours (3 AM-7 AM) where the risk is 2 to 3 times higher than that at other times of the day. The relative risk of nocturnal hypoglycemia compared with daytime hypoglycemia was greater in patients with HI than in those with IKH. This risk is greater still in patients with HI above the age of 10 months, those with genetic mutations, and those off medication.

A strength of this study is the novel examination of a glycemic phenotype using CGM in patients with HI while using an alternative model of hypoglycemia in children with IKH as a contrasting paradigm, demonstrating the specificity of glycemic profiles within each condition’s digital phenotype. Although home blood glucose monitoring remains the standard of care for monitoring in patients with HI, we have used high-granularity glucose data to expand the phenotype of HI and highlight an important role for CGM, that is, describing nocturnal glycemic status in real time with high ecological validity. Alternative strategies to identify periods of hypoglycemia on the basis of parent interviews would be open to recall bias and unable to identify unexpected hypoglycemia, as self-monitoring of blood glucose is rarely performed overnight. With increasing refinements in CGM technology and the increasing popularity of the use of CGM in children with hypoglycemia, our study highlights the need for a targeted application of CGM. Although previous studies have used simplistic correlation methods to test the accuracy of CGM in the detection of hypoglycemia vis-à-vis home blood glucose monitoring, our study has investigated a deeper phenotype with significant clinical impact.

Our study is exploratory, as a similar analysis of CGM data has never been attempted. Therefore, it is not possible to validate the strength of our observations, except that the glycemic phenotype is replicable across the whole group with HI, in contrast to an alternative model of hypoglycemia in IKH. The number of patients with HI was large for a rare disease, and the total number of measurements in the data set adds strength to the rigor of the study.

It is not possible to investigate the cause of early hours hypoglycemia from the design of our study, nor was this the intended purpose. We speculate that the unavailability of carbohydrates due to the nocturnal fasting period, high glucose demand in the brain at this time [23,31], and the suppression of counter-regulatory gluconeogenesis in patients with HI [30] could be the probable causes. The latter view is supported by the observation of a reduced tendency to early hours hypoglycemia in patients with IKH and an increased tendency in those positive for genetic mutations known to cause HI. Patients with IKH do not have underlying metabolic disturbances and are therefore capable of mounting adequate counter-regulatory responses. In contrast, those positive for HI mutations tend to have more severe disease [11] and less ability to mount counter-regulatory responses. Further investigation of the metabolic and counter-regulatory hormonal milieu in patients with HI may be required to refine specific causation. It is not clear why patients negative for mutations had a relatively high incidence of hypoglycemia between 9 PM and 1 AM, and further work will be required to investigate this apparent trend.

We observed a tendency for early hours hypoglycemia in patients with HI above the age of 10 months (Figure 4); this may be explained by older children sleeping longer and requiring less frequent feeds through the night. We did not record sleep-wake cycles in our study to confirm or refute this possibility. However, children below the age of 10 months were also more likely to be inpatients during the period of monitoring, and the lack of early hours hypoglycemia tendency may reflect 24-hour nursing care and a reduction in ecological validity. The reason for the significant increase in time spent in hypoglycemia between 7 PM and 8 PM is unclear and may reflect the small number of patients from whom this sample was taken. Further data are required to validate or refute this finding.

Patients with HI who were no longer receiving HI medications showed a clear tendency toward hypoglycemia during early hours seen in other groups. This response was not observed in patients receiving medications for HI (diazoxide or octreotide). This may reflect the efficacy of HI medications in preventing nocturnal hypoglycemia but may also be simply reflective of the much higher median age in the off-treatment group and the subsequent effects described above. Because this study was completed, it was not possible to assess the relative impacts of age and medication on the timing of hypoglycemia in patients with HI, and further work is required.

Tendency to hypoglycemia was shown to reduce throughout the day in patients with HI, but with small increase in risk at 9 AM, 3 PM, and 7 PM (Figure 3). These periods of increased risk are likely to correlate with postmeal times in those having three meals a day, suggesting possible postprandial hypoglycemia secondary to the hyperinsulinemic response to food sometimes observed in patients with HI [46]. This trend was not observed in patients with IKH who had fasting rather than postprandial hypoglycemia. This was not observed in patients with HI below the age of 10 months who would have been receiving a high proportion of caloric intake as milk feeds distributed more frequently throughout the day and night.

As hypoglycemia thresholds were reduced from 3.9 mmol/L to 3.5 mmol/L and 3.0 mmol/L, the number of hypoglycemic events and total minutes spent hypoglycemic also reduced significantly, as reflected in the differing Y axes in Figures S5 and S6 (Multimedia Appendix 3). This is unsurprising, as patients with HI are told to maintain glucose levels >3.5 mmol/L and those with IKH >3.0 mmol/L. Reducing the hypoglycemia threshold emphasized the tendency for early hours hypoglycemia in those with HI and allowed it to become apparent in those with IKH. These likely better reflect the true hypoglycemic events that would be acted on by parents.

There were 42 hypoglycemia events lasting more than 30 minutes in patients with HI despite them wearing an unblinded CGM device programmed to alarm at glucose levels <3.5 mmol/L and parents being aware of the importance of keeping glucose levels ≥3.5 mmol/L at all times. This high prevalence of prolonged and potentially dangerous hypoglycemia events may reflect CGM inaccuracy, and these events may represent false positives not acted on by parents. However, this is unlikely to explain all the prolonged hypoglycemic events.

Although CGM provides vital information, the volume of data provided by continuous monitoring such as CGM can be overwhelming for both health care professionals and patients [47], and simple detection and reporting of glucose values will not be sufficient to eliminate all hypoglycemia. It is well recognized that alarm fatigue is a significant problem [48] and that only 37% of parents will wake to hypoglycemia alarms that sound overnight [49]. Other behavioral explanations are possible, including voluntary alarm switching off and leaving the receiver out of earshot. The engagement of parents with the CGM device and their behaviors in response to data provided comprise a vital extension of the digital phenotype of HI [40,42].

Future work must further evaluate this aspect of the digital phenotype of HI to better understand not only the underlying pathophysiology but also the human in the loop. Simple mathematical modeling to generate ever better glycemic predictions is unable to eradicate hypoglycemic events in the real world if it does not factor in how human behaviors change in response to the data.

### Conclusion

We provide the first analysis of the timing of hypoglycemia in patients with hypoglycemia due to HI using CGM data and, in doing so, expand the digital phenotype. In contrast to the phenotype of hypoglycemia in children with IKH, a clear period of high risk for hypoglycemia was observed in patients with HI in the early hours (3 AM-7 AM). Such early hours hypoglycemia was particularly frequent in those with genetic mutations known to cause HI and in children older than 10 months. Despite the unblinded state-of-the-art technology, prolonged and potentially harmful hypoglycemic events were detected in patients with HI. Hypoglycemia during early hours poses a high risk for neuroglycopenic brain injury, and behavioral aspects of the digital phenotype in HI must be evaluated so that interventions can be designed to maximize effect.

## Appendix

Multimedia Appendix 1 Notepad file with original Python code used for data analysis. This can also be found freely available on GitHub [<xref ref-type="bibr" rid="ref49">49</xref>].

Multimedia Appendix 2 Further hyperinsulinism subgroup comparisons.

Multimedia Appendix 3 Comparison of reducing hypoglycemia thresholds.

## Abbreviations

CGM: continuous glucose monitoring
HI: hyperinsulinism
IKH: idiopathic ketotic hypoglycemia
