# Supplementary material for: Clustering of Hypoglycemia Events in Patients With Hyperinsulinism: Extension of the Digital Phenotype Through Retrospective Data Analysis
Source: J Med Internet Res. 2021 Oct 29;23(10):e26957. doi: 10.2196/26957 (PMC8590184; doi:10.2196/26957)
Supplement: Multimedia Appendix 2 [file jmir_v23i10e26957_app2.doc]

# Multimedia Appendix 2: further HI subgroup comparisons

The tendency to early hours hypoglycemia was preserved in those datasets from HI patients who tested positive for a mutation (n = 16) in genes known to cause hyperinsulinism (*ABCC8 (n = 13)*, *GCK (n = 1)* and *SLC16A1 (n = 2)*) (Figure S1) but less clearly in those who either tested negative (n = 5) or were not tested (n = 2) (Figure S2). In the mutation positive group, risk of early hours hypoglycemia was 6.7% (1135/17005 minutes) compared with a 2.8% (290/81810) risk outside the early hours (Chi-square 78.1, *P* < .001). This indicates that those with severe forms of HI due to gene mutations had a greater tendency to nocturnal hypoglycemia. For those in the mutation negative/not tested group, risk of early hours vs other time (day/night) hypoglycemia was less pronounced but still significant (5.9% (530/8870) vs 3.1% (1295/41680), Chi-square 21.5, *P* < .001), indicating that the tendency to nocturnal hypoglycemia was regulated by aetiology wider than genetic influence alone.

Figure S1. Percentage time hypoglycemic by hour of the day in mutation positive HI patients. *Analysis of timings of hypoglycemia in those HI patients positive for a mutation in genes known to cause HI demonstrates a clear tendency to early hours hypoglycemia*


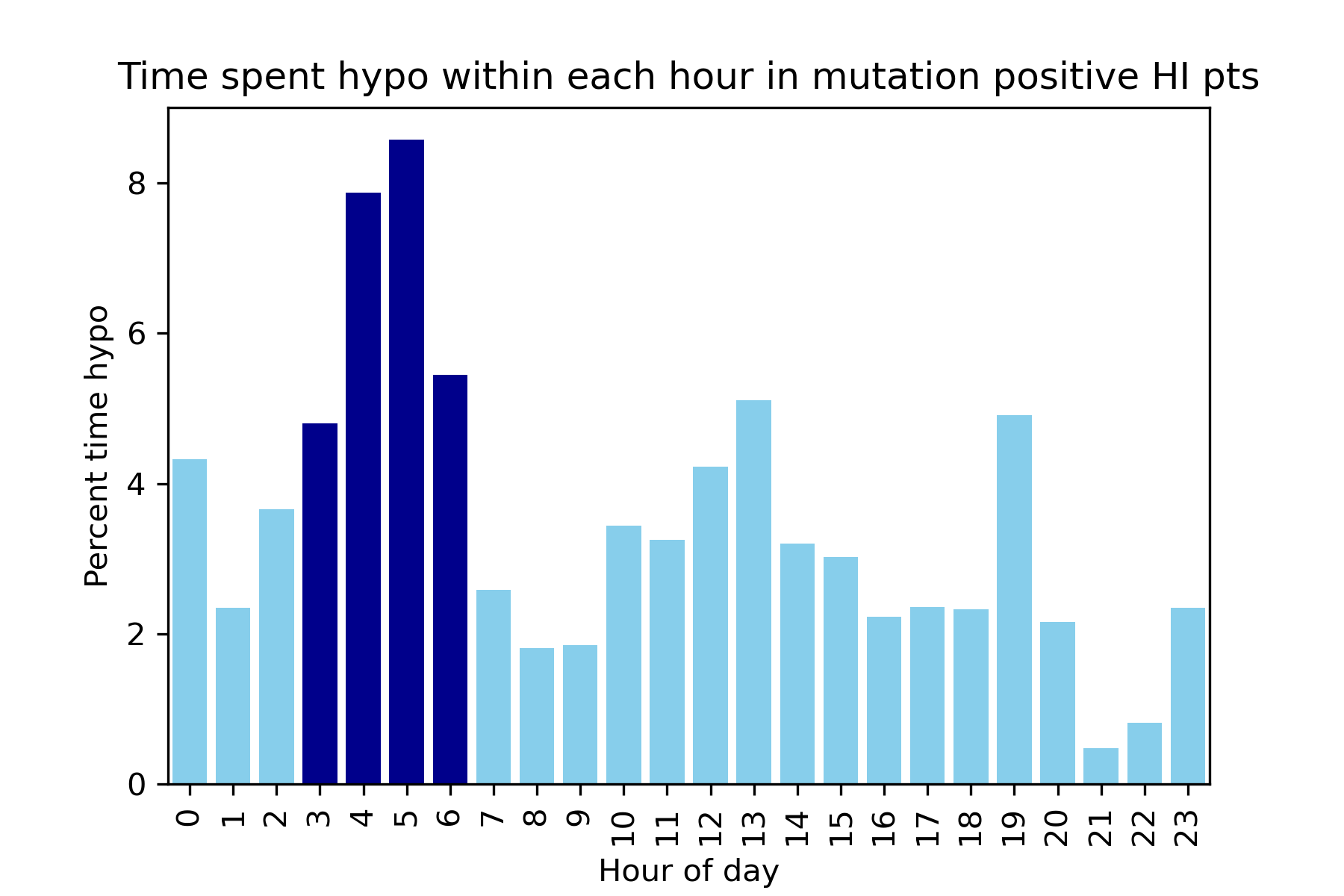


Figure S2. Percentage time hypoglycemic by hour of the day in mutation negative HI patients. *Analysis of timings of hypoglycemia in those HI patients negative or not tested for a mutation in genes known to cause HI demonstrates less of tendency to early hours (dark blue) hypoglycemia than in other groups.*

*
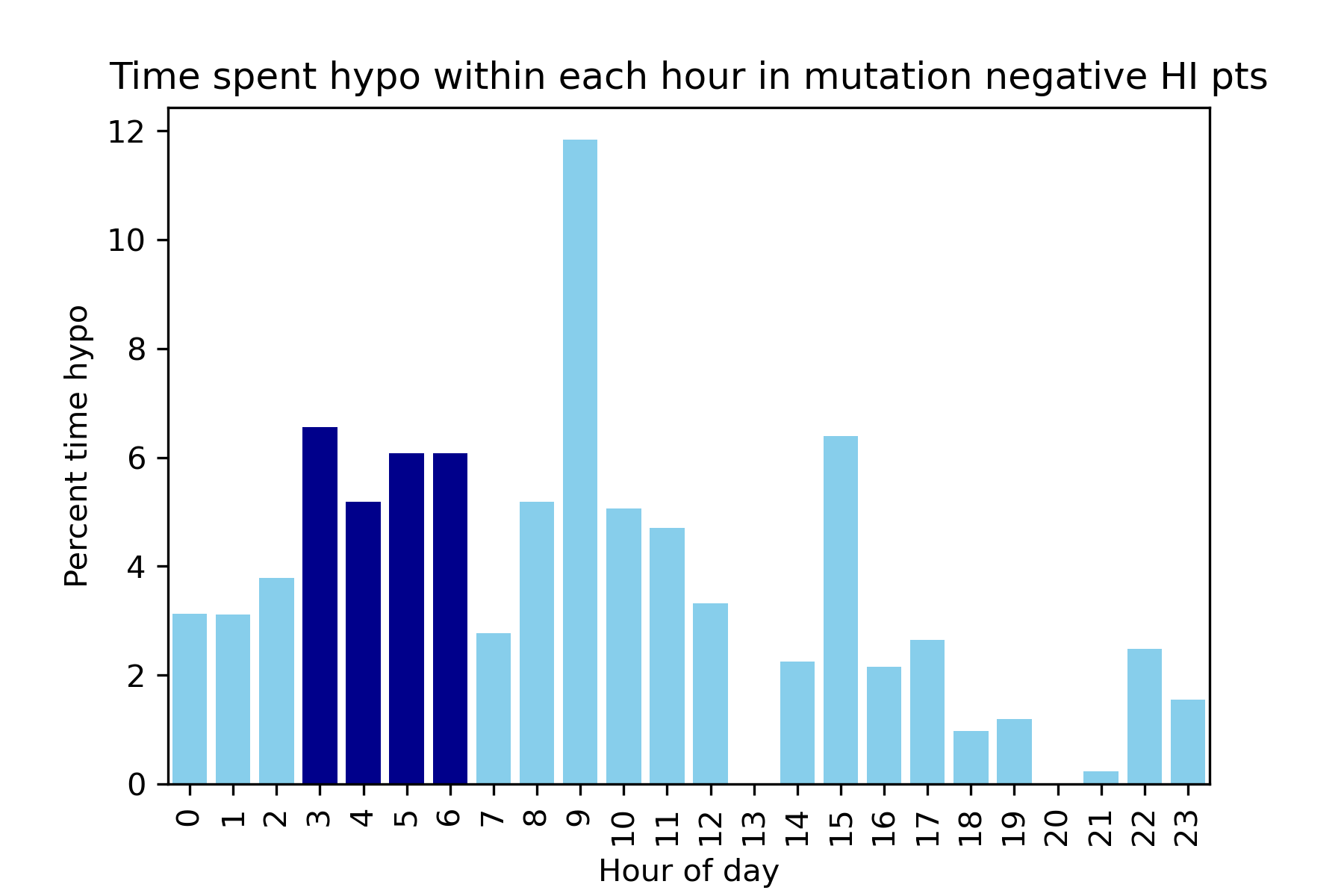
*

The exaggerated tendency to early hours hypoglycemia in the mutation positive group was not explained by an older age at investigation. In the mutation positive group, age (months) was marginally lower than the mutation negative/not tested group (49 vs 73, *P* = .213) and there was greater frequency of cases in the younger (< 10 month) subcategory (6/7 vs 1/7, *P* = .172).

Timing of hypoglycemia was also tested for differences between those on and off medication (diazoxide or octreotide). Those patients off medication displayed a much clearer tendency towards early hours hypoglycemia (Figure S3) than those still on medication (Figure S4). In those off medication, 32% of all hypoglycemia was in the early hours compared with only 18% in those on medication. If hypoglycemia was distributed entirely evenly throughout the day then one would expect 16% of hypoglycemia in these hours. There was a difference in mean age (months) between the off and on medications groups (80 vs 36, *P* = 0.079.

Figure S3. Percentage of time spent hypoglycemic by hour of the day in HI patients off medication. *A clear tendency to early hours (dark blue) hypoglycemia is seen.*


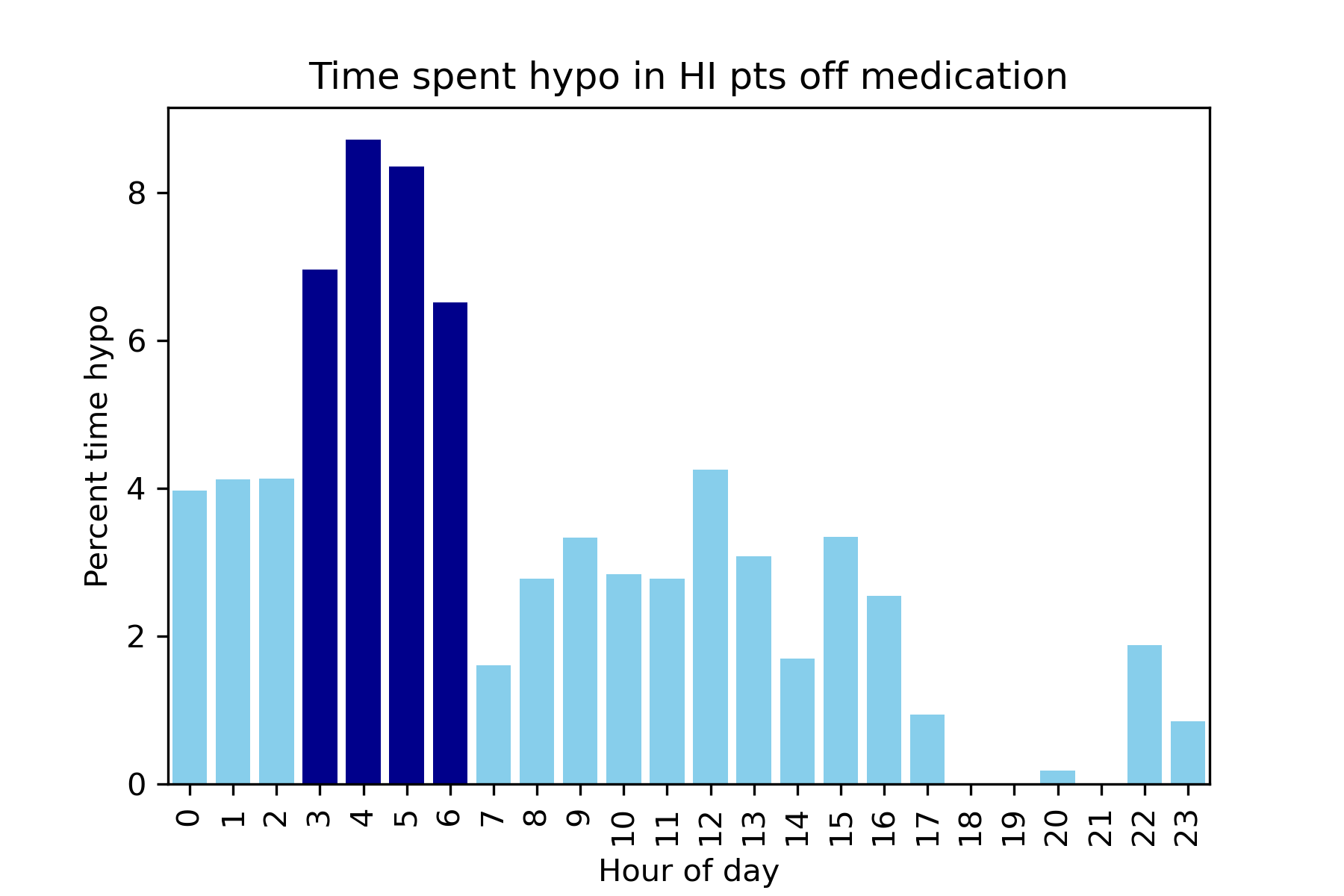


Figure S4. percentage of time spent hypoglycemic by hour of the day in HI patient on medication. *The early hours tendency to hypoglycemia seen in those off medications is not replicated in those still on medications.*


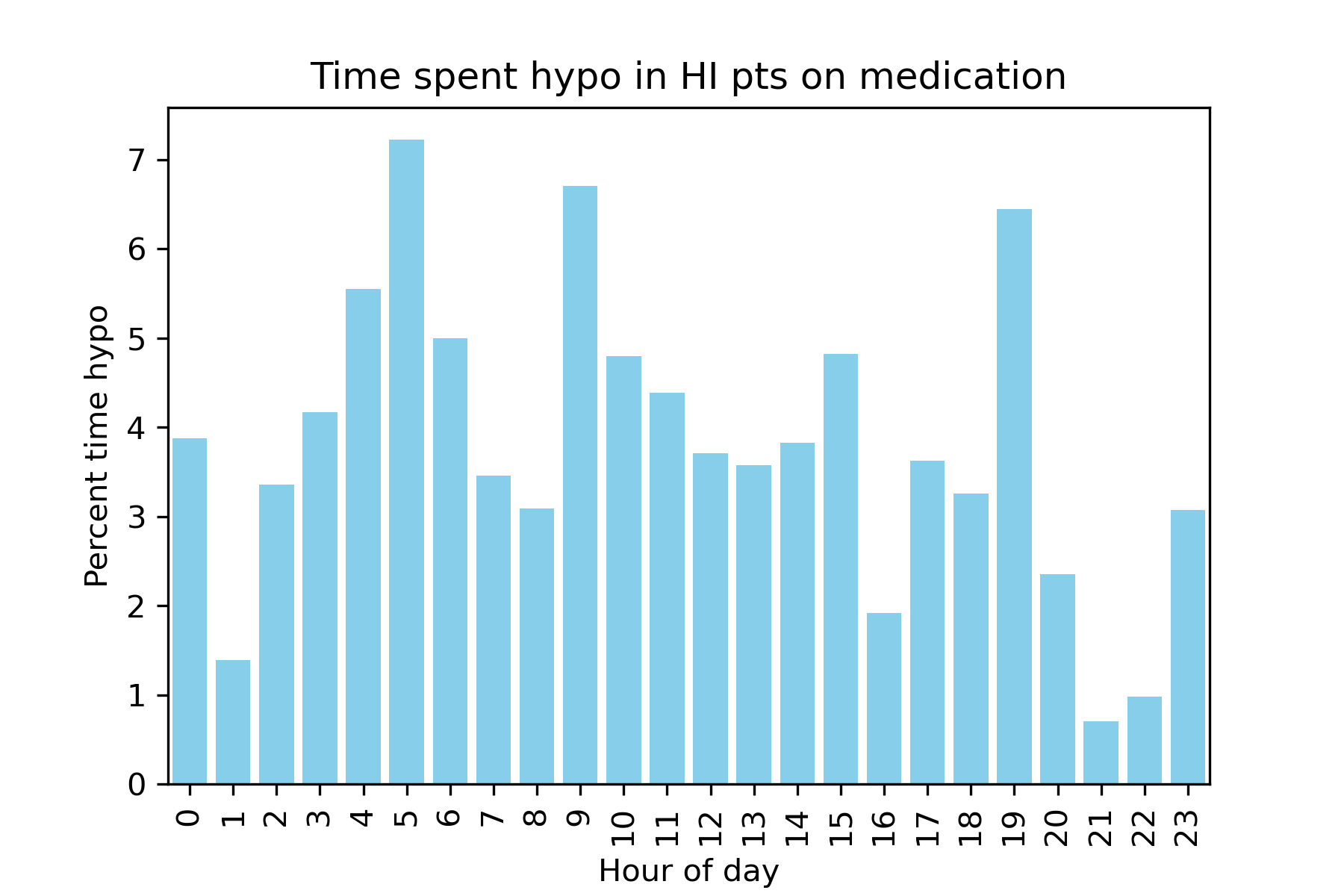


Further analyses of subgroups were undertaken between focal vs diffuse HI. Visualisations and statistics are not reported for these subgroups as there was a significantly different mean age (months) (7 vs 79, *P* < .001) and any differences are likely a direct reflection of the differences found between the groups under and over 10 months of age.
