# Supplementary material for: Clustering of Hypoglycemia Events in Patients With Hyperinsulinism: Extension of the Digital Phenotype Through Retrospective Data Analysis
Source: J Med Internet Res. 2021 Oct 29;23(10):e26957. doi: 10.2196/26957 (PMC8590184; doi:10.2196/26957)

# Multimedia Appendix 3: Figures S5 and S6

Figure S5. Percentage time hypoglycemic by hour of the day in HI patients > 10 months of age with differing thresholds of hypoglycemia. *Lowering the threshold of hypoglycemia from 3.9mmol/L to 3.5mmol/L and 3.0mmol/L demonstrates the persistence of the early hours as the period of highest risk for hypoglycemia.*


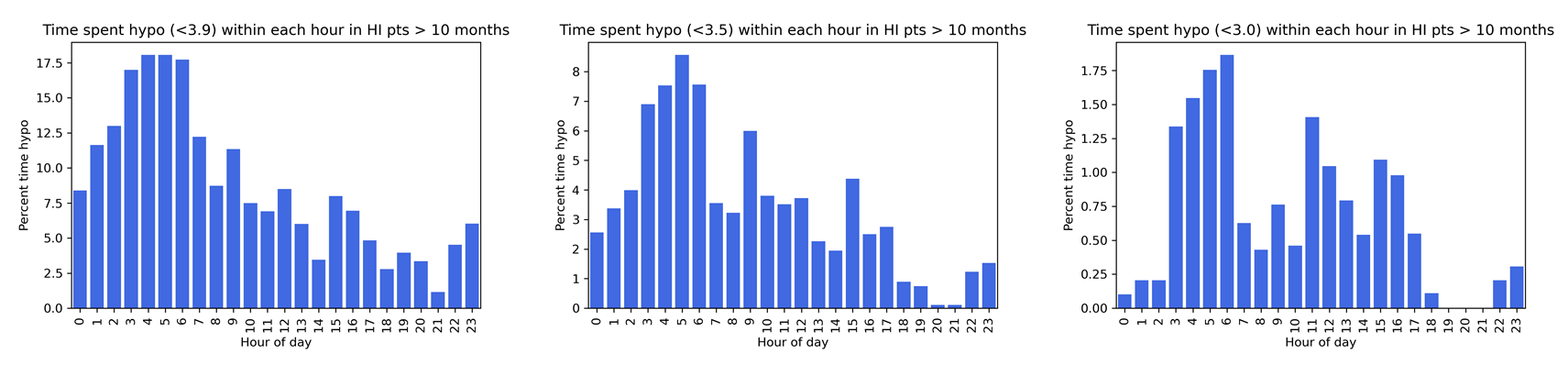


Figure S6. Percentage time hypoglycemic by hour of the day in IKH patients with differing thresholds of hypoglycemia. *Lowering the threshold of hypoglycemia from 3.9mmol/L to 3.5mmol/L and 3.0mmol/L allows for the emergence of the early hours as the period of highest risk of hypoglycemia in this cohort.*


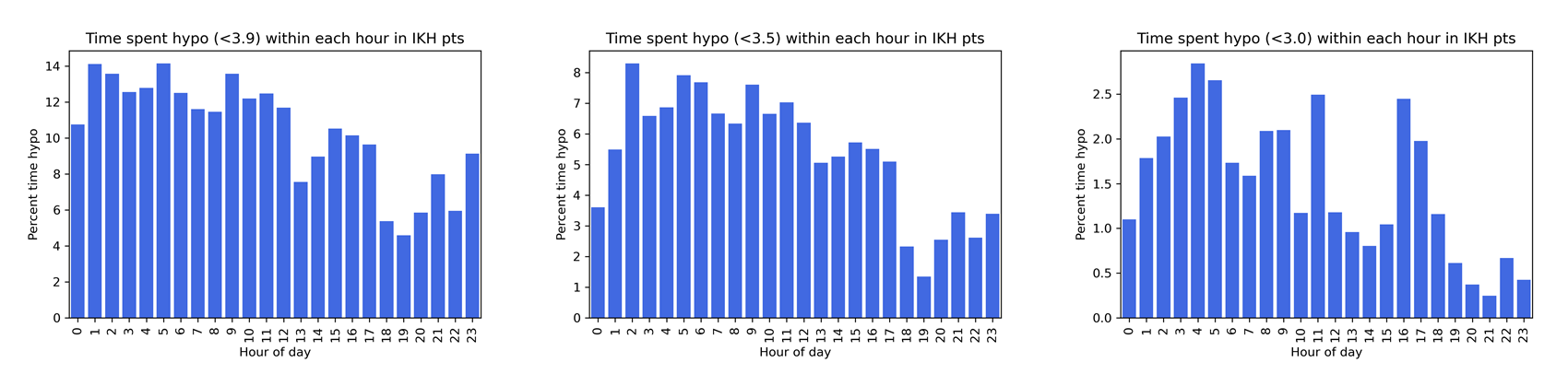

Supplement: Multimedia Appendix 3 [file jmir_v23i10e26957_app3.doc]
